# The effect of two endogenous retinoids on the mRNA expression profile in human primary keratinocytes, focusing on genes causing autosomal recessive congenital ichthyosis

**DOI:** 10.1007/s00403-014-1476-4

**Published:** 2014-06-13

**Authors:** H. Törmä, A. Bergström, G. Ghiasifarahani, B. Berne

**Affiliations:** 1 Department of Medical Sciences, Dermatology and Venereology, Uppsala University, SE-751 85 Uppsala, Sweden; 2 Science for Life Laboratory, Uppsala University, SE-751 85 Uppsala, Sweden

**Keywords:** All-*trans* retinoic acid, 3,4-didehydroretinoic acid, MicroRNA, Keratin, Retinoic acid receptors

## Abstract

Retinoids (natural forms and synthetic derivatives of vitamin A) are used as therapeutic agents for numerous skin diseases such as keratinization disorders (e.g. ichthyoses) and psoriasis. Two endogenous ligands for retinoic acid receptors exist, retinoic acid (atRA) and 3,4-didehydroretinoic acid (ddRA). In primary human epidermal keratinocytes many transcriptional targets for atRA are known, whereas the targets for ddRA are unknown. In an attempt to determine the targets, we compared the effect of atRA and ddRA on transcriptional profiles in undifferentiated and differentiating human primary keratinocytes. First, as expected, many genes were induced or suppressed in response to keratinocyte differentiation. Furthermore, the two retinoids affected substantially more genes in differentiated keratinocytes (>350) than in proliferating keratinocytes (≈20). In differentiating keratinocytes markers of cornification were suppressed suggesting a de-differentiating effect by the two retinoids. When comparing the expression profile of atRA to that of ddRA, no differently regulated genes were found. The array analysis also found that a minor number of miRNAs and a large number of non-coding transcripts were changed during differentiation and in response to the two retinoids. Furthermore, the expression of all, except one, genes known to cause autosomal recessive congenital ichthyosis (ARCI) were found to be induced by differentiation. These results comprehensively document that atRA and ddRA exert similar transcriptional changes in keratinocytes and also add new insights into the molecular mechanism influenced by retinoids in the epidermis. Furthermore, it suggests which ARCI patients could benefit from therapy with retinoids.

**Electronic supplementary material:**

The online version of this article (doi:10.1007/s00403-014-1476-4) contains supplementary material, which is available to authorized users.

## Introduction

Retinoids (natural and synthetic forms of vitamin A) have profound influence on normal keratinocytes both in vitro and in vivo, the best-known effect probably being the inhibition of cellular differentiation. This has resulted in the use of retinoids as therapeutic agents for skin diseases with disturbed keratinization, i.e., ichthyoses and psoriasis [35, 53]. The effects of endogenous vitamin A (retinol) are mediated by its metabolites all-*trans* retinoic acid (atRA) and 3,4-didehydroretinoic acid (ddRA) [34]. In vitro, retinol is metabolized to atRA and ddRA primarily in differentiated keratinocytes [34, 51]. The keratinocyte-specific ddRA has seemingly similar effects as atRA in some assays [49].

atRA and ddRA exert most of their biological effects by binding to and activating nuclear retinoic acid receptors (RARα, -β and -γ). A second class of retinoid receptors, RXRs (RXRα, -β and -γ), functions as heterodimerization partner for RARs (for review see [30]). These RAR–RXR heterodimers bind to RA-response elements (RAREs) in the promoter region of specific genes (for review see [2]), which lead to altered transcription of the adjacent gene. Several of the retinoid receptors are expressed by human epidermal keratinocytes. The most abundant forms in falling order are RXRα, RARγ and RARα [15], thus making RARγ/RXRα the predominant heterodimer. According to some studies, these receptors are expressed mainly by cells in differentiated cell layers of normal epidermis [25, 36], suggesting that they are involved in the process of terminal differentiation. Yet, another function of unliganded RARs is in the formation of lipid-containing lamellar granules in terminally differentiated keratinocytes [11, 16].

Retinoids also interact with other signaling pathways, e.g., the activator protein-1 (AP-1) (for review see [2]). AP-1 is a regulator of genes induced by differentiation of keratinocytes, e.g., transglutaminase 1 (*TGM1*), loricrin (*LOR*), keratin 1 (*KRT1*), and involucrin (*IVL*) (see [13] for review), all of which are repressed by atRA [7, 18, 37]. It has also been shown that the DNA-binding activity of AP-1 is increased in cultured keratinocytes induced to differentiate by elevated extracellular calcium [24].

The transcription of many genes is regulated by atRA and a list of 532 genes as regulatory targets of atRA was compiled by evaluating 1,191 published papers [1]. Transcriptional profiling studies in response to atRA have been performed in epidermal keratinocytes and reconstructed skin [4, 26].

The aim of this study was to examine whether the two endogenous retinoids atRA and ddRA affect the mRNA expression profile differently in cultured human primary keratinocytes using oligonucleotide arrays. All genes causing ARCI were analyzed in detail. The expression profiles after retinoid treatment were examined both in proliferating and differentiated keratinocytes, as representatives for basal and suprabasal epidermal keratinocytes, respectively.

## Materials and methods

### Materials

atRA and ddRA were obtained from Sigma-Aldrich (Stockholm, Sweden) and Roche AG (Basle, Switzerland), respectively. The compounds were dissolved in ethanol and the concentration and purity were determined by UV absorbance and HPLC analysis, respectively. Stock solutions of 1 mM were prepared. The purity of atRA and ddRA was 99.1 and 88.3 %, respectively. The two major impurities in ddRA were 13cRA (1.5 %) and atRA (10.2 %). All handling of retinoids was performed under yellow reduced light.

### Cell culture and retinoid treatment

Primary keratinocytes from a lightly pigmented female donor (42 years) were used at passage 3 (Life Technologies, Stockholm, Sweden). The cells were maintained in EpiLife serum-free keratinocyte medium containing growth supplement (HKGS or HKGS kit) and gentamicin/amphotericin (all from Life Technologies). Proliferating cells in 6-well plates at 30–40 % confluency were switched to EpiLife medium without EGF for 5 h prior to starting treatment with retinoids. Keratinocytes at 80 % confluency were switched to EpiLife^−^ supplemented with 1.5 mM CaCl_2_ for 4 days with the purpose to induce cellular differentiation. Cells were subsequently exposed to retinoids as above in EpiLife medium without rhEGF. Both proliferating and differentiated cells were exposed to 1 µM of atRA, ddRA or vehicle (ethanol) for 4 and 24 h. All treatments were performed in triplicates. The cells were washed with PBS twice and frozen at −70 ^°^C until further processing.

### Microarray expression analysis

The cells were dissolved in 1 ml TriReagent (Ambion, Life Technologies, Carlsbad, CA), and total RNA was isolated as previously described [50]. RNA concentration was measured with ND-1000 spectrophotometer (NanoDrop Technologies, Wilmington, DE), and RNA quality was evaluated using the Agilent 2100 Bioanalyzer system (Agilent Technologies Inc, Palo Alto, CA). Total RNA (250 ng) from each sample was used to generate amplified and biotinylated sense-strand cDNA from the entire expressed genome according to the GeneChip^®^ WT PLUS Reagent Kit User Manual (P/N 703174 Rev 1 Affymetrix Inc., Santa Clara, CA). GeneChip^®^ ST Arrays (GeneChip^®^ XXX Gene 2.1 ST Array) were hybridized for 16 h in a 45 °C incubator, washed and stained and finally scanned at the GeneTitan^®^ Multi-Channel (MC) Instrument, according to the GeneTitan Instrument User Guide for Expression Arrays Plates (PN 702933 Rev 2., Affymetrix Inc., Santa Clara, CA).

### Microarray data analysis

The raw data were normalized in the free software Expression Console provided by Affymetrix (http://www.affymetrix.com) using the robust multi-array average (RMA) method [21, 27]. The RMA algorithm fits a robust linear model at the probe level to minimize the effect of probe-specific affinity differences. Subsequent analysis of the gene expression data was carried out in the freely available statistical computing language R (http://www.r-project.org) using packages available from the Bioconductor project (www.bioconductor.org). In order to search for the differentially expressed genes between different groups, an empirical Bayes' moderated *t* test was then applied [40], using the ‘limma’ package [41]. To address the problem with multiple testing, the *p* values were adjusted using the method of Benjamini and Hochberg [3].

Heat maps of gene expression were generated using Genesis gene expression similarity investigation suite [44]. Genes were considered regulated if the expression levels differed more than 1.5-fold relative to controls and had an adjusted *p* value of <0.05. Functional annotation of regulated genes was performed as before using the Database for Annotation, Visualization and Integrated Discovery (DAVID) [19, 20]. Gene ontology clusters with a *p* value <10^−4^ were retained for evaluation. The complete data are submitted to Gene Expression Omnibus repository (in process).

### Analysis of mRNA expression using quantitative PCR

First-strand cDNA was synthesized from 1.5 µg total RNA by combining oligo(d)T_15_, random hexamers, buffer and MMLV reverse transcriptase (Life Technologies) as previously described [50].

Semi-quantitative PCR was performed using cDNA (10 ng total RNA) as template and TaqMan Gene Expression Assays and TaqMan Universal PCR Master Mix, No AmpErase UNG in an ABI7500Fast PCR machine (Life Technologies). TaqMan gene expression assays were used to detect *TGM1*, *PNPLA1, LRAT, KRT2,* and *KRT4*. Expression levels were measured in duplicate.

The following genes were analyzed with SYBR Green detection: *PPIA-*encoding cyclophilin A (used as reference genes for geNorm normalization [54]), *TGM1*, *INL*, *HB*-*EGF*, *ALOX12B*, and *SCCE*. Analysis was performed using an ABI PRISM 7500Fast sequence detection system (Applied Biosystems) as described elsewhere [8, 9]. The relative mRNA expression was determined by the 2^(−ΔΔCt)^ method and analyzed by one-way ANOVA followed by Tukey’s multiple comparison test (Prism 5.04 software, GraphPad Software Inc., La Jolla, CA).

## Results

### Comparison between differentiating and proliferating primary keratinocytes

We compared the transcriptional profiles of vehicle-exposed differentiating and proliferating keratinocytes using GeneChip^®^ XXX Gene 2.1 ST Arrays. The microarrays simultaneously measure the levels of >41,000 coding and alternative splicing transcript variants and microRNA (miRNA) and long intergenic non-coding transcripts. Of these, approximately ~25,500 coding transcripts, 1,170 miRNA and 17,900 non-coding transcripts were found to be expressed in keratinocytes. Among them were 709 induced and 730 suppressed in differentiating cells, according to our criteria (Table 1). The expressed genes in differentiating and proliferating cells are shown as a heat map (Online Resource 1), and the top ten-induced and -suppressed genes are given in Online Resource 2. Of the induced transcripts were 577 coding and 6 miRNA, and of the suppressed transcripts were 606 coding and 10 miRNA, the remaining transcripts in both groups were non-coding transcripts. Among the many genes affected were 11 genes belonging to the keratin family. *KRT1, KRT2, KRT4, KRT6B, KRT6C, KRT10, KRT23, KRT77,* and *KRT80* were all induced, whereas *KRT15* and *KRT16* were suppressed. Many of the genes induced by differentiation are also known to be mutated in autosomal recessive congenital ichthyosis (ARCI) or syndromes with congenital ichthyosis, i.e., *TGM1, ALOX12B, ALOXE3, CYP4F22, NIPAL4, ABCA12, PNPLA1, CERS3, ABHD5, ALDH3A2*, *ELOVL4* and *SPINK5* (Table 2). One exception was *SLC27A4*, encoding FATP4.

**Table 1:** Altered coding transcripts, miRNA and non-coding RNAs in differentiating vs. proliferating keratinocytes and the effect of exposure to atRA and ddRA for 4 and 24 h

	Differentiating vs. proliferating	Differentiating keratinocytes				Proliferating keratinocytes			
	24 h	4 h		24 h		4 h		24 h	
		atRA vs. vehicle	ddRA vs. vehicle	atRA vs. vehicle	ddRA vs. vehicle	atRA vs. vehicle	ddRA vs. vehicle	atRA vs. vehicle	ddRA vs. vehicle
Induced									
>2 FC	424	39	28	86	77	3	3	8	7
1.5–1.99 FC	285	45	55	121	98	4	3	5	8
Transcripts	709	84	83	207	175	7	6	13	15
Suppressed									
>2 FC	324	4	5	69	97	0	0	1	1
1.5–1.99 FC	406	11	8	98	107	1	2	5	9
Transcripts	730	15	13	167	204	1	2	6	10

Induced and suppressed transcripts showing a fold change (FC) >2, and transcripts with a fold change in the range 1.5–1.99 are reported in two separate groups

*Diff.* differentiating keratinocytes, *Prol.* proliferating keratinocytes, *atRA* all-trans retinoic acid, *ddRA* 3,4-didehydroretinoic acid

**Table 2:** Expression of genes causing autosomal recessive congenital ichthyosis and syndromes with ichthyosis in cultured keratinocytes

Gene	Differentiating vs. proliferating		Differentiating	
	Vehicle vs. vehicle (24 h)		atRA vs. vehicle (24 h)	
	Fold change	Adjusted *p* value	Fold change	Adjusted *p* value
*ALOX12B*	5.07	7.14 E-17	−2.66	4.69 E-10
*TGM1*	4.47	2.67 E-21	−0.99	7.45 E-07
*LIPN*	4.46	1.01 E-15	−2.55	8.63 E-10
*PNPLA1*	3.29	1.67 E-18	−1.88	3.16 E-12
*ABCA12*	2.89	5.60 E-23	−0.01	0.974
*NIPAL4*	2.86	2.20 E-17	−0.50	0.004
*CYP4F22*	2.81	4.99 E-13	−0.69	0.011
*CERS3*	2.00	1.02 E-17	−0.33	0.006
*ALOXE3*	1.97	4.66 E-14	−1.34	7.60 E-10
*SLC27A4*	1.16	9.49 E-11	0.04	0.869
*ABHD5*	1.68	1.62 E-15	−0.76	1.03 E-07
*ELOVL4*	2.42	6.75 E-19	−0.51	1.00 E-04
*SPINK5*	5.24	2.69 E-21	0.33	0.174
*ALDH3A2*	1.77	1.05 E-16	−0.73	6.76 E-08
*GBA*	0.83	6.21 E-05	−0.19	0.550

The effect of differentiation and exposure to all-*trans* retinoic acid. Genes were considered regulated if the expression levels differed more than 1.5-fold relative to controls and had an adjusted *p* value of <0.05

**Fig. 1:**
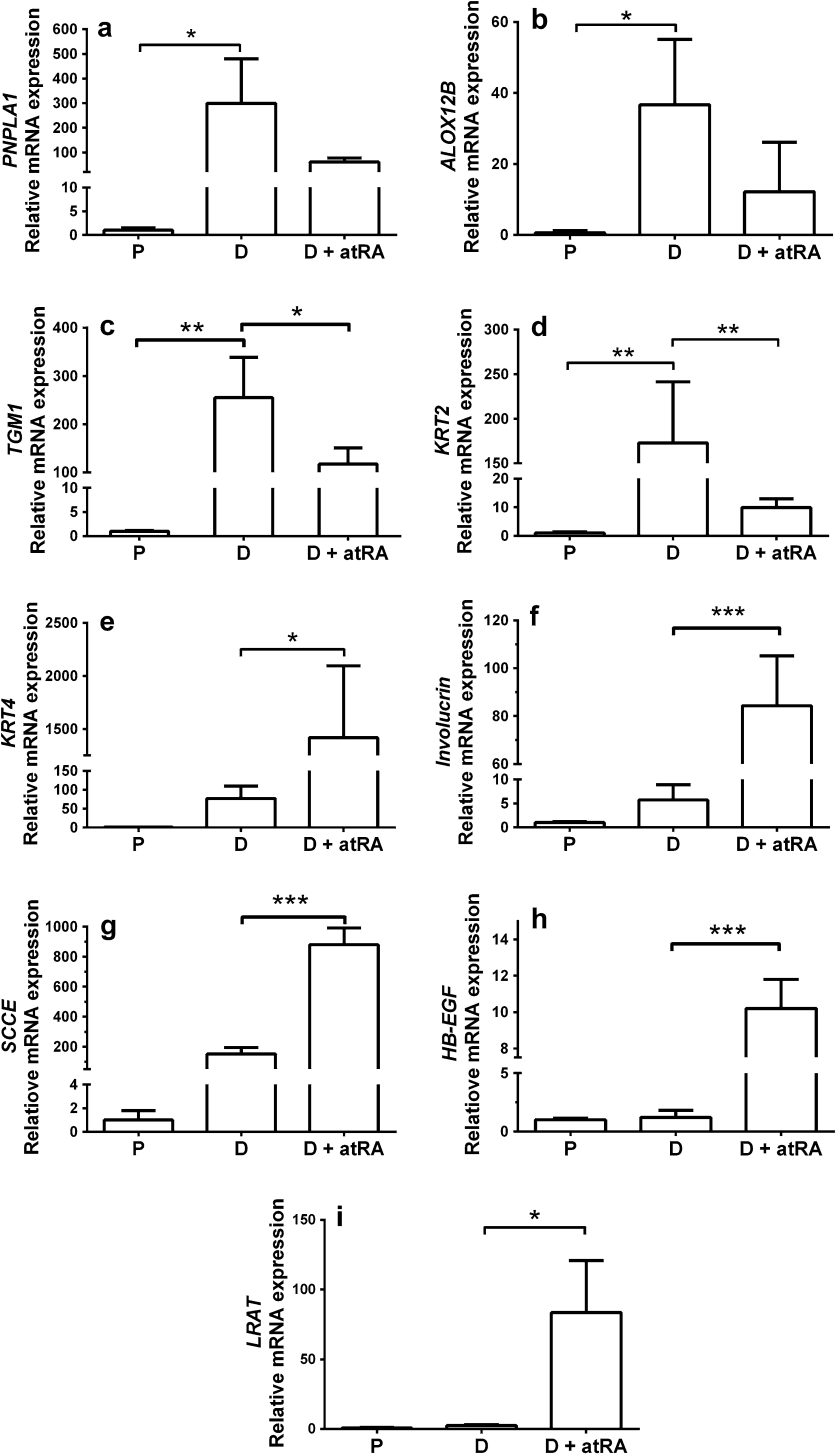
Analysis of mRNA expression in proliferating, differentiating and atRA-treated differentiating keratinocytes by quantitative polymerase chain reaction. The mRNA expression of **a** *PNPLA1,* **b** *ALOX12B,* **c** *TGM1,* **d** *KRT2,* **e** *KRT4,* **f** *INV,* **g** *SCCE,* **h** *HB*-*EGF* and **i** *LRAT* was examined. The expression of *PNPLA1*, *ALOX12B*, *TGM1* was found to be induced in differentiating vs. proliferating keratinocytes. The expression of *TGM1* and *KRT2* was significantly reduced in response to atRA treatment in differentiating keratinocytes, whereas the expression of *KRT4, INV, SCCE, HB*-*EGF* and *LRAT* was induced. **a**–**h** * *p* < 0.05, ** *p* < 0.01, * *p* < 0.001

To confirm the oligonucleotide array results obtained, we performed quantitative RT-PCR analysis of genes altered in differentiating vs. proliferating cells or by atRA exposure in differentiating cells. The analysis verified the changes in the mRNA profiling in differentiating vs. proliferating cells (Fig. 1a–c).

Certain miRNAs were also induced (i.e., *MIR203, MIR4451, MIR3671, MIR27B, MIR23B*) and suppressed (i.e., *MIR1305, MIR4435*-*1, MIR29A, MIR622, MIR181B1, MIRLET7A2, MIR424, MIR503*) upon differentiation.

### Functional characterization of the genes regulated by differentiation

Functional annotation clustering analysis was performed using DAVID on the genes regulated by differentiation. We found that more than 21 % of the induced genes fall into one of the following ontological categories of biological processes: epidermis development, cell junction, plasma membrane, membrane fraction, cellular lipid metabolic process (Online Resource 3). Among the suppressed genes more than 20 % of them were annotated to the regulation of cell proliferation, blood vessel development, DNA metabolic process, regulation of programmed cell death and regulation of cell cycle.

### atRA and ddRA regulate gene expression most profoundly in differentiating keratinocytes

We compared the transcriptional profiles in atRA- and ddRA-treated keratinocytes with vehicle-treated keratinocytes. In cells exposed to one of the two retinoids, the affected RNA transcripts were few in numbers in proliferating keratinocytes as compared to differentiated cells (see Table 1). The genes altered in response to atRA exposure is shown as a heat map (Online Resource 4). However, the number of altered transcripts was in the same range after treatments with both retinoids, and most of the transcripts were significantly altered by both retinoids. The top ten-induced and -suppressed annotated transcripts after atRA exposure are shown in (Online Resource 5). The expression of some genes in atRA- vs. vehicle-treated differentiating cells was verified by qPCR (Fig. 1b–i).

Many of the 151 annotated transcripts induced in differentiating keratinocytes are known to be retinoid regulated, e.g., *STRA6, BMP6, LRAT, KRT13, KRT19, KRT23, KRT31, KRT4, DHRS3, DHRS9, KLK6*, *HB-EGF, S100A7 (PSORIASIN)* and *PI3 (SKALP)*. Among the ones not previously reported were *GBP6, GPR176, SERPINB1, SERPINB3, STAT4,* and *CLEC2*. Some of the 143 annotated transcripts suppressed in differentiating keratinocytes are known to be affected by retinoids, e.g., *KRT1, KRT2*, *FLG* and *TGM1*. The effect on the expression of ARCI-causing genes is given in Table 2. Among the ones not previously reported were *GPR155, CDHR1, FLG2,* and *ACER1*. There was also altered expression of a few miRNAs. MIR1305 and MIR3975 were induced and MIR203, MIR4774, MIR3671, and MIR4451 were suppressed by atRA exposure.

### Functional characterization of the atRA-regulated genes in differentiating keratinocytes

When we summarized the functional categories of the genes up-regulated by atRA at 24 h in differentiated keratinocytes, we found that 11 % of them fall into the categories tissue development or hormone metabolic process (Online Resource 6). Among the suppressed genes, more than 10 % of them were annotated to either lipid catabolic process or vesicular fraction.

When combining all up-regulated and suppressed genes about 40 % of the genes were related to plasma membrane, endoplasmic reticulum, cell–cell junction, endopeptidase activity and intermediate filament (Online Resource 6).

### Gene expression was not regulated differently by atRA and ddRA

Although the major number of retinoid-regulated transcripts was induced or suppressed equally efficiently by atRA and ddRA, there were also transcripts that were significantly altered by only one of the retinoids when comparing to vehicle-treated controls. However, when comparing the expression profiles generated by the two retinoids with each other, no genes were significantly altered (data not shown).

## Discussion

In the present study, we examined gene expression profiles in cultured keratinocytes. First, we examined the expression profiles in proliferating and differentiating keratinocytes and secondly, the effects of exogenous addition of two retinoids which normally are synthesized by epidermal keratinocytes, atRA and ddRA. The reason for comparing the expression profile in both cell types is that retinoid receptors are to a major extent expressed by keratinocytes in suprabasal layers of epidermis [25, 36], suggesting that retinoids may affect gene transcription mainly in differentiated cells.

We found that the genes altered by differentiation were involved in epidermis development, plasma membrane, cellular lipid metabolic process, regulation of cell proliferation, blood vessel development, DNA metabolic process and regulation of DNA metabolic process. Our results verify the results by others regarding many of the altered genes [26]. Several of the genes are regulated during keratinocyte differentiation [26, 45].

Using a macro-array methodology, it has been shown that retinoids generate different effects when added to reconstructed human epidermis as compared to cultured keratinocytes [4]. In these experiments, the cultured cells were not induced to differentiate and thus the cells are equivalent to the proliferating cells in the present experiments.

Others have found that atRA suppresses markers of cornification and genes involved in de novo lipogenesis [26]. Also, at RA was shown to regulate the pathways of its own bioavailability and genes associated with the cell cycle and programmed cell death [26]. In the present study, we found regulation of pathways related to cornification genes, few genes involved in atRA bioavailability and programmed cell death but not of genes involved in de novo lipogenesis. The lack of effect on the expression of these genes could be due to difference between the experiments with respect to the number of time points investigated and the culture conditions (proliferating vs. differentiating) [26]. In the present study, differentiation was achieved by increasing calcium concentration for the last 3 days and removing EGF for the last 5 h prior to addition of retinoids to the medium.

Autosomal recessive congenital ichthyosis is to date known to be caused by ≥10 genes [46]. Many of these genes are involved in transport or metabolism of epidermal lipids, whereas other genes are involved in modifying omega-hydroxy-glucosylceramides for covalent binding to the cornified lipid envelope, a step which is performed by transglutaminase-1 (encoded by *TGM1*) [14]. We found several of the genes causing ARCI and other forms of ichthyosis to be induced during keratinocyte differentiation, i.e., *TGM1, ALOXE3, ALOX12B, NIPAL4, CYP4F22, ABCA12, ABHD5, PNPLA1, ELOVL4*, *LIPN* and *CERS3* (see Table 2). The present results correlate well with the previously reported expression in cultured keratinocytes [12, 23, 31, 42] and localization of respective protein in upper layers of normal epidermis [10, 17, 28, 29, 33, 38, 39]. However, to the best of our knowledge, this is the first time the expression of *ALOX12B, ALOXE3, LIPN, CYP4F22, PNPLA1* and *ELOVL4* has been studied in cultured human keratinocytes. Surprisingly, *SLC27A4* transcripts were not induced by differentiation. Furthermore, some of the transcripts were significantly suppressed by retinoids, i.e., *ALOX12B*, *PNPLA1,* and *LIPN* (see Table 2).

It was recently shown that keratinocyte differentiation is positively regulated by the microRNA mir-203 [43]. MIR203 (and MIR3671) were induced by differentiation in our array analysis supporting the robustness of the present data, with respect to miRNAs. Furthermore, both MIR203 and MIR3671 expressions were suppressed by atRA exposure in differentiated keratinocytes. This suggests that also mir-3671 might be a positive regulator of keratinocyte differentiation. On the other hand, mir-1305 expression was altered in an opposite manner compared to these two miRNAs.

The role of 3,4-didehydroretinoids (3,4-didehydroretinol, 3,4-didehydroretinaldehyde and 3,4-didehydroretinoic acid) is unclear. In mouse keratinocytes, it has been shown that apoptosis is induced to greater extent by ddRA compared to atRA [22]. This effect on apoptosis is supported by induction of genes annotated to apoptosis after exposure to atRA as previously described [26]. However, in the present study no difference in transcription profiles was found between atRA and ddRA at any time point.

It is possible that these two endogenous retinoids do not have different functions or that one should look at different aspects of biological function, not directly related to gene transcription. In other organisms, 3,4-didehydroretinaldehyde, alone or in combination with retinaldehyde, is used in the retinal chromophore [32, 47, 48, 52]. In Gecko lizards it has been shown that in the eye lens CRBPI has 3,4-didehydroretinol as ligand which gives the lens a yellow color [55]. It has been suggested that 3,4-didehydroretinoids in the retina diminishes the harmful effects of short-wave radiation [55]. Furthermore, in human and rabbit skin it has been shown that exposure to UVB light degrades retinol, but not 3,4-didehydroretinol [5, 6]. This may suggest that the biosynthesis of epidermal 3,4-didehydroretinoids serves as a back-up system for retinol and retinoic acid during situations of exposure to UV radiation.

In summary, these data present a comprehensive description of the transcriptional changes caused by atRA and ddRA treatment of proliferating and differentiating primary human keratinocytes. The transcriptional changes obtained by the two retinoids were exactly the same, ruling out different transcriptional functions for these two endogenous retinoids formed by epidermal keratinocytes. We also found that the expression of 13 genes causing ARCI and syndromes with ichthyosis is induced in differentiating keratinocytes, and four of these genes showed reduced expression upon atRA treatment. The effect of retinoids in the treatment of ARCI patients could thus be due to inhibition of the expression of the disease-causing genes or a general effect on keratinocyte differentiation. Surprisingly, far from all ARCI-causing genes were suppressed by atRA exposure arguing against a de-differentiation effect. Hypothetically, responders to retinoid therapy might be found among those patients with mutations in genes that are not affected by retinoids in vitro (due to suppression of residual enzymatic activity).

## Electronic supplementary material

Below is the link to the electronic supplementary material.

Supplementary material 1 (PDF 69 kb)

Supplementary material 2 (JPEG 2496 kb)

Supplementary material 3 (JPEG 3065 kb)
