# Supplementary material for: The effect of two endogenous retinoids on the mRNA expression profile in human primary keratinocytes, focusing on genes causing autosomal recessive congenital ichthyosis
Source: Arch Dermatol Res. 2014 Jun 13;306(8):739–47. doi: 10.1007/s00403-014-1476-4 (PMC4168020; doi:10.1007/s00403-014-1476-4)
Supplement: Supplementary file 1 — Supplementary material 1 (PDF 69 kb) [file 403_2014_1476_MOESM1_ESM.pdf]

“The effect of two endogenous retinoids on the mRNA expression profile in human primary keratinocytes, focusing on genes causing autosomal recessive congenital ichthyosis”
Archives of Dermatological Research
H. Törmä, A. Bergström, G. Ghiasifarahani, B. Berne
Uppsala University, Sweden. e-mail:hans.torma@medsci.uu.se

Online Resource 2

Top ten induced and suppressed genes in response to differentiation.

Induced	Fold change	p-value		Suppressed	Fold change	p-value
MUC15	7,275	3,38E-21		ANXA6	-4,837	1,08E-23
DAPL1	6,955	9,06E-18		EMP3	-4,654	4,04E-20
KRT1	6,744	5,26E-21		ADAMTS1	-4,468	6,27E-23
DSC1	6,413	3,38E-21		MIG7	-4,133	2,96E-09
ZNF750	6,246	1,31E-19		MT1L	-3,973	1,35E-13
C10orf99	6,074	2,19E-21		HS3ST2	-3,964	5,26E-21
THEM5	6,067	3,40E-19		VIM	-3,932	9,95E-23
SERPINB4	5,995	4,01E-15		RPSAP52	-3,914	3,54E-14
KRTDAP	5,937	3,23E-21		PTHLH	-3,904	1,77E-24
SBSN	5,920	6,68E-27		POSTN	-3,899	8,24E-21

“The effect of two endogenous retinoids on the mRNA expression profile in human primary keratinocytes, focusing on genes causing autosomal recessive congenital ichthyosis”
Archives of Dermatological Research
H. Törmä, A. Bergström, G. Ghiasifarahani, B. Berne
Uppsala University, Sweden. e-mail:hans.torma@medsci.uu.se

Online resource 3.

Functional annotation of regulated genes (differentiating versus proliferating cells) was performed using the Database for Annotation, Visualization and Integrated Discovery (DAVID) with the most significant P-value listed. For a complete list of all the clusters with a P-value less than 10^{-4}, see Supplemental Table 2. Genes showing a 1.5-fold change was included.

**Clusters induced**	**#**	**p-value**
epidermis development	35	5.72E-20
plasma membrane part	87	7.87E-5
cellular lipid metabolic process	32	2.62E-5
**Clusters suppressed**		
regulation of cell proliferation	56	6.59E-9
blood vessel development	24	1.87E-6
DNA metabolic process	52	4.03E-14
regulation of DNA metabolic process	14	4.54E-5
**Clusters (all upregulated and suppressed genes)**		
epidermis development	42	2.73E-14
regulation of cell proliferation	82	1.32E-7
cell-cell junction	31	1.84E-7
endoplasmic reticulum	86	9.16E-6
regulation of cell differentiation	55	2.77E-6
DNA replication	31	2.74E-7

#The number of genes in each category.

"The effect of two endogenous retinoids on the mRNA expression profile in human primary keratinocytes, focusing on genes causing autosomal recessive congenital ichthyosis"
Archives of Dermatological Research
H. Törmä, A. Bergström, G. Ghiasifarahani, B. Berne
Uppsala University, Sweden. e-mail:hans.torma@medsci.uu.se

Online Resource 5

The top ten induced and suppressed genes in response to atRA-exposure in differentiated keratinocytes. Cultured keratinocytes were differentiated for four days by omitting rhEGF and adding 1.2 mM calcium chloride to the culture medium for four days. Retinoic acid (1 µM) was added for 24h.

Induced	Fold change	Adjusted p-value		Suppressed	Fold change	Adjusted p-value
CEACAM6	5,22	4,13E-12		C10orf99	-5,40	2,01E-19
IL1B	4,92	2,24E-26		CASP14	-4,66	2,30E-18
PAPPA	4,77	5,29E-19		THEM5	-4,60	1,61E-15
KLK6	4,40	1,17E-21		KRT2	-4,60	1,93E-16
BHLHE41	4,30	2,01E-19		DAPL1	-4,57	7,41E-13
STRA6	4,17	3,47E-16		METTL7A	-4,04	2,29E-15
BMP6	4,09	2,92E-17		AADACL2	-3,77	8,20E-13
TCN1	4,08	8,96E-18		ANKRD35	-3,68	1,26E-13
RHCG	4,07	1,67E-18		CLEC2A	-3,66	3,83E-14
CALB2	4,01	4,77E-12		ACER1	-3,62	2,41E-15

“The effect of two endogenous retinoids on the mRNA expression profile in human primary keratinocytes, focusing on genes causing autosomal recessive congenital ichthyosis”
Archives of Dermatological Research
H. Törmä, A. Bergström, G. Ghiasifarahani, B. Berne
Uppsala University, Sweden. e-mail:hans.torma@medsci.uu.se

Online Resource 6

Functional annotation of regulated genes (atRA- versus vehicle-treated cells) in differentiating keratinocytes to biological processes was performed using the Database for Annotation, Visualization and Integrated Discovery (DAVID) with the most significant P-value listed. Genes showing a 1.5-fold change was included.

**Clusters induced**	**#**	**p-value**
tissue development	16	8.82E-5
hormone metabolic process	7	1.53E-4
**Clusters suppressed**		
lipid catabolic process	10	1.49E-6
vesicular fraction	8	6.72E-4
**Clusters (up-regulated and suppressed)**		
plasma membrane	80	7.56E-5
endoplasmic reticulum	30	9.88E-5
cell-cell junction	12	6.62E-5
endopeptidase activity	16	3.61E-4
intermediate filament	11	2.28E-4

The number of genes in each category.
