# Supplementary figures and images for: The effect of two endogenous retinoids on the mRNA expression profile in human primary keratinocytes, focusing on genes causing autosomal recessive congenital ichthyosis
Source: Arch Dermatol Res. 2014 Jun 13;306(8):739–47. doi: 10.1007/s00403-014-1476-4 (PMC4168020; doi:10.1007/s00403-014-1476-4)

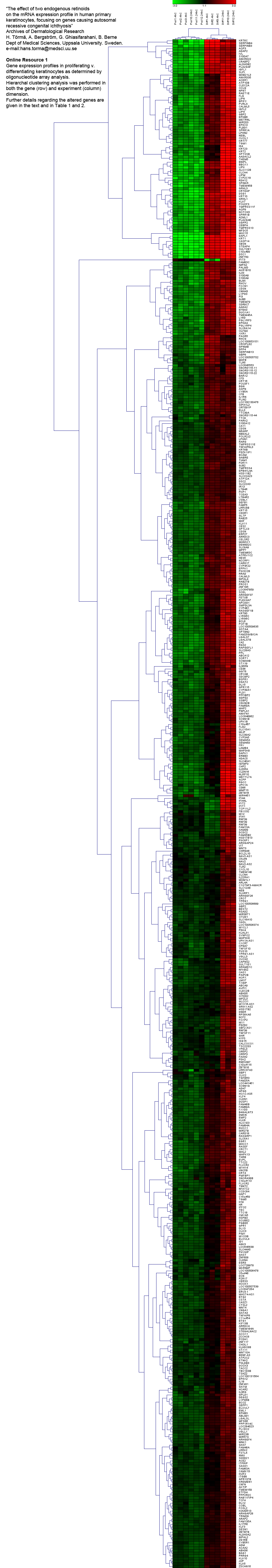

Supplement: Supplementary file 2 — Supplementary material 2 (JPEG 2496 kb) [file 403_2014_1476_MOESM2_ESM.jpg]

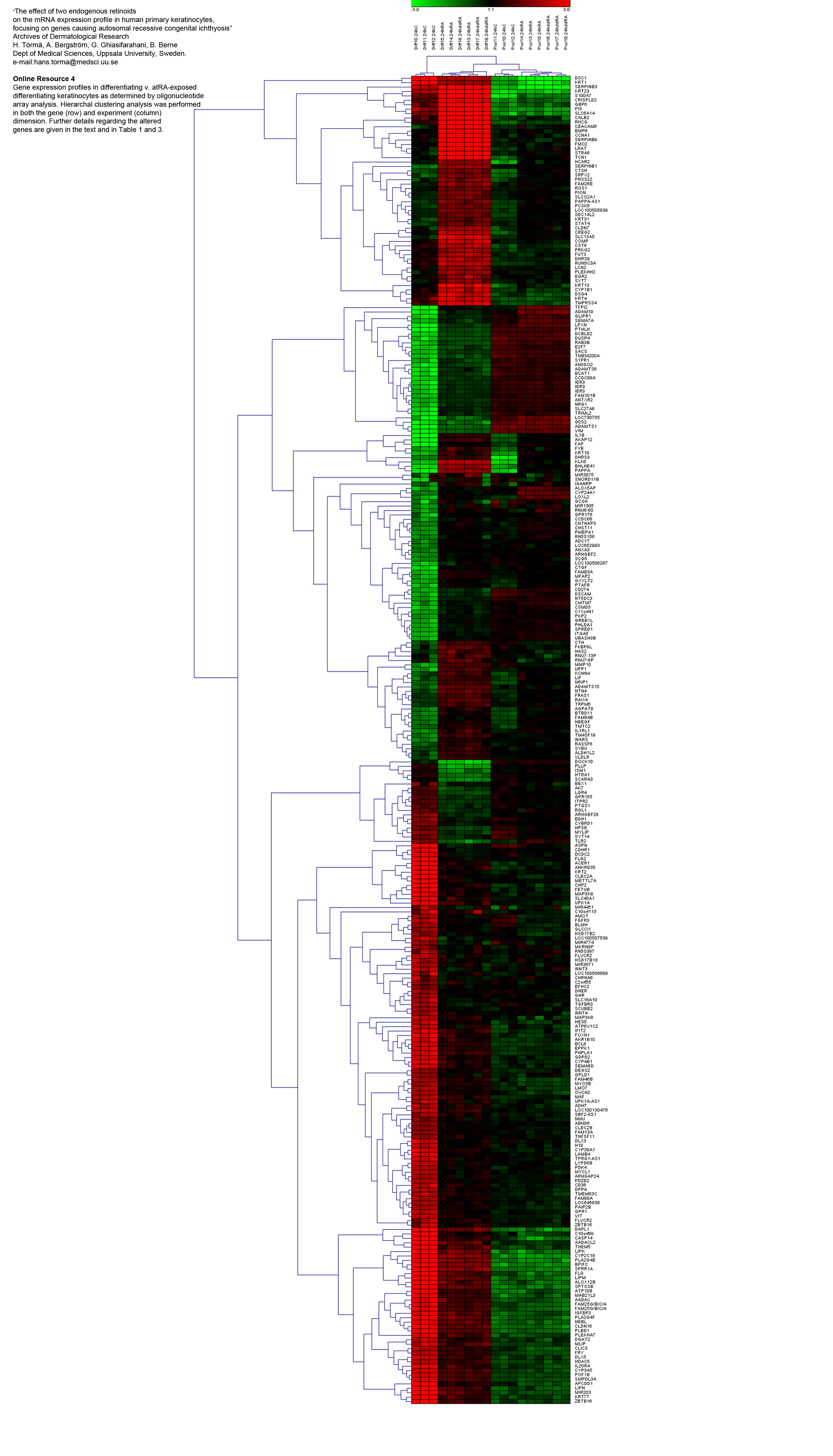

Supplement: Supplementary file 3 — Supplementary material 3 (JPEG 3065 kb) [file 403_2014_1476_MOESM3_ESM.jpg]
